# Submetric Spatial Resolution ROTDR Temperature Sensor Assisted by Wiener Deconvolution

**DOI:** 10.3390/s22249942

**Published:** 2022-12-16

**Authors:** Wenhao Zhu, Haoting Wu, Weixuan Chen, Meiting Zhou, Guolu Yin, Nan Guo, Tao Zhu

**Affiliations:** 1 The Key Laboratory of Optoelectronic Technology & Systems (Ministry of Education), Chongqing University, Chongqing 400044, China; 2 State Key Laboratory of Coal Mine Disaster Dynamics and Control, Chongqing University, Chongqing 400044, China

**Keywords:** distributed temperature sensing, ROTDR, Wiener deconvolution, spatial resolution

## Abstract

A submetric spatial resolution Raman optical time-domain reflectometry (ROTDR) temperature sensor assisted by the Wiener deconvolution postprocessing algorithm has been proposed and experimentally demonstrated. Without modifying the typical configuration of the ROTDR sensor and the adopted pump pulse width, the Wiener demodulation algorithm is able to recover temperature perturbations of a smaller spatial scale by deconvoluting the acquired Stokes and anti-Stokes signals. Numerical simulations have been conducted to analyze the spatial resolution achieved by the algorithm. Assisted by the algorithm, a typical ROTDR sensor adopting pump pulses of 20 ns width can realize the distributed temperature sensing with a spatial resolution of 0.5 m and temperature accuracy of 1.99 °C over a 2.1-km sensing fiber.

## 1. Introduction

Distributed optical fiber sensing (DOFS) has received wide research attention and has been applied in temperature sensing [1,2,3], strain sensing [4,5,6], and gas detection [7,8]. Based on the types of backscattered light, DOFS can be divided into Rayleigh optical fiber sensing [9,10], Brillouin optical fiber sensing [11,12,13], and Raman optical fiber sensing [14,15,16]. Compared to the other two techniques, Raman optical fiber sensing is only sensitive to the ambient temperature of the sensing fiber and is able to avoid crosstalk from fiber strains or vibrations. Moreover, Raman optical time-domain reflectometry (ROTDR) offers a low-cost solution due to its simple system configuration. Thanks to these features, the technique is attractive in many fields, such as pipeline leakage detection [17], heatsink temperature monitoring [18], and early fire warning [19].

The finite signal-to-noise ratio (SNR) of the ROTDR scheme requires the adopted pump pulses of over several tens of nanoseconds. This is one of the dominant factors limiting its spatial resolution [15,17,19], making the technique less suitable for many applications. Researchers have contributed several innovative works in recent years to break through the meter scale spatial resolution of the technique. A reconstruction compression correlation demodulation scheme was proposed as a possible solution to realize a spatial resolution of 7.5 mm [20]. Unfortunately, the limited bandwidth of today’s photodetectors keeps this scheme from experimental demonstration. Alternatively, based on an efficient polarization-independent superconducting nanowire single-photon detector, a ROTDR scheme with a spatial resolution of 10 cm is experimentally demonstrated on a 500-m fiber [21]. However, the method employs pump pulses of hundreds of picoseconds, which means that the energy of each pump pulse is low, and the temperature accuracy is difficult to ensure. A slope-assisted scheme is also proposed to realize a centimeter-level spatial resolution [22]. However, slope detection involves second-order differentiation, which significantly amplifies the inherently high noise level of the system and leads to a relatively inferior temperature accuracy.

Deconvolution is a processing technique to estimate or recover the desired process from the recorded degraded process. As one of the most classic deconvolution approaches, Wiener deconvolution based on Wiener filtering has been widely employed for 1-D signal recovery [23] and 2-D image deblurring from the recorded degraded signals or images with noise [24]. In 1996, Nakamura et al. reported a spatial resolution of 0.5 m at 1 km in the ROTDR system. [25] However, they didn’t disclose the details of their deconvolution method or provide any numerical simulations. In 2009, Zhang et al. realized a spatial resolution of 15 m with a 300-ns pulse width with Wiener deconvolution [26]. However, the resulting 15-m spatial resolution is relatively inferior for today’s applications, and the best spatial resolution achieved by the algorithm is not studied sufficiently. In 2016, Bazzo et al. improved the spatial resolution of a commercial ROTDR sensor system (AP Sensing N4385B) from 1 m to 15 cm based on total variation deconvolution algorithm [27], which requires a much more complicated iteration operation. Recently, the Wiener deconvolution technique has been introduced into the DOFS area to enhance the spatial resolution of typical Brillouin optical time-domain reflectometry (BOTDR) [28]. In this work, the postprocessing algorithm based on the Wiener deconvolution technique is researched to further enhance the spatial resolution of a typical ROTDR scheme from meter scale to submetric scale. The influence of the deconvolution parameter of the Wiener deconvolution algorithm and the bandwidth of the modulation and detection modules on the spatial resolution after deconvolution are studied numerically. The Wiener deconvolution is experimentally demonstrated to process the Raman scattering signals obtained from a typical ROTDR sensing system by using 20-ns width pump pulses, and realizes the distributed temperature sensing with a spatial resolution of 0.5 m and temperature accuracy of 1.99 °C over a 2.1-km sensing fiber.

## 2. Principles and Simulations

**Figure 1:**
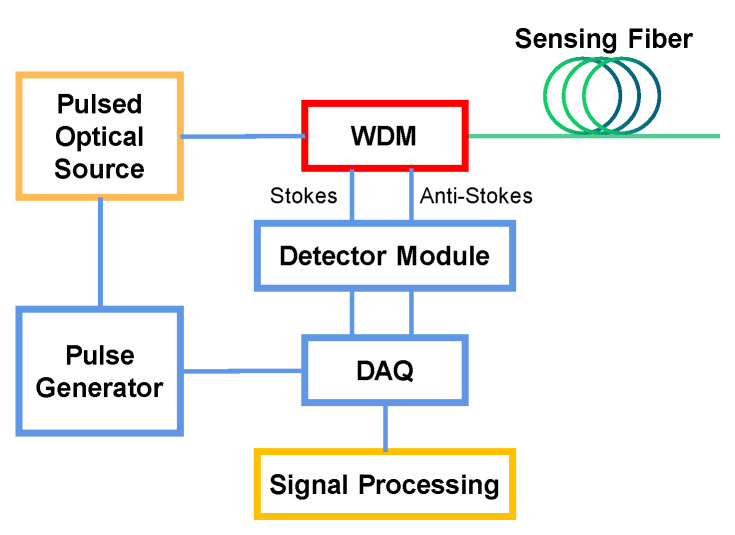
Schematic configuration of a generic ROTDR temperature sensor system. WDM, wavelength division multiplexer; DAQ, data acquisition.

Figure 1 shows the schematic configuration of a generic ROTDR temperature sensor system. High-power optical pulses are launched into the sensing fiber via a wavelength division multiplexer (WDM). Backscattered anti-Stokes and Stokes Raman lightwaves are guided into the detection module and converted to electrical signals, which are collected by a data acquisition (DAQ) module and then processed to extract the temperature distribution.

The temperature distribution along the sensing fiber is extracted from the ratio of the Raman anti-Stokes signal over Raman Stokes responses as $A\left(z\right)=I_{\mathrm{AS}}\left(2z/v_{g}\right)/I_{S}\left(2z/v_{g}\right)$, where $I_{\mathrm{AS}}\left(t\right)$ and $I_{S}\left(t\right)$ are, respectively, the anti-Stokes and Stokes Raman signals, $v_{g}$ is the group velocity of light, and *z* is location distance along the sensing fiber. The ratio is dominated by [29]

(1) $$A\left(z\right)=\frac{K_{\mathrm{AS}}\nu_{\mathrm{AS}}^{4}}{K_{S}\nu_{S}^{4}}\exp\left(-\frac{h\Delta \nu }{kT(z)}\right)\exp\left[\left(\alpha_{S}-\alpha_{\mathrm{AS}}\right)z\right],$$

where T(z) is the absolute temperature at location distance of *z*, $K_{\mathrm{AS}}$ and $K_{S}$ are related to the anti-Stokes and Stokes Raman backscattering section, $\nu_{\mathrm{AS}}$ and $\nu_{S}$ are the frequency of anti-Stokes and Stokes Raman backscattering photons, $\alpha_{\mathrm{AS}}$, $\alpha_{S}$ are the attenuation of anti-Stokes Raman light and Stokes Raman light, *h* is the Planck’s constant, *k* is the Boltzmann’s constant, Δν is the Raman frequency shift. If the temperature distribution changes from the reference temperature $T_{0}\left(z\right)$ to T(z),

(2) $$T\left(z\right)=\frac{h\Delta \nu T_{0}\left(z\right)}{h\Delta \nu -kT_{0}\left(z\right)ln\left[A\left(z\right)/A_{0}\left(z\right)\right]},$$

where $A_{0}\left(z\right)$ is the calibrated value of the Raman anti-Stokes/Stokes ratio at location *z* under temperature of $T_{0}$.

The output current signals of the detection module $I_{\mathrm{AS}}\left(t\right)$ and $I_{S}\left(t\right)$ can be expressed as [29]

(3) $$\begin{array}{cc} I_{†}\left(t\right) & =R_{†}\left(t\right)*p\left(t\right)*g_{†}\left(t\right)+n_{†}\left(t\right)\\ & =\psi_{†}\left(t\right)*g_{†}\left(t\right)+n_{†}\left(t\right) \end{array},$$

where $R_{†}\left(t\right)$ is the impulse response function of the detection module, p(t) is the pump pulse envelope profile, $g_{†}\left(t\right)$ is the original ideal Raman response signal, $n_{†}\left(t\right)$ is the additive system noise, the operator * represents convolution, and the subscript † is used to represent AS or S donating anti-Stokes and Stokes Raman light components, respectively. The convolution indicates that the original ideal Raman response signal $g_{†}\left(t\right)$ is degraded by $\psi_{†}\left(t\right)$, which may submerge the high-frequency signal fluctuations of the recorded Raman signals induced by the temperature change within a short fiber spatial segment. As the temperature distribution is typically obtained by the ratio of the recorded Raman anti-Stokes and Stokes signals, the convolutions lead to the rising/falling edge of the received Raman signals and therefore the measurement ambiguity in the spatial scalar. The ambiguity is basically presented as the transition area of the temperature distribution curve, whose length is commonly characterized as the spatial resolution of the sensor system. The expression of the degradation function $\psi_{†}\left(t\right)=R_{†}\left(t\right)*p\left(t\right)$ given in Equation (3) means the function can be calibrated by using the identical detection module to detect the pump pulse. It turns out that $\psi_{\mathrm{AS}}\left(t\right)$ and $\psi_{S}\left(t\right)$ share almost the same normalized envelope profile. Therefore, the acquired pump pulse signal after normalization can be regarded as the degradation function for the recorded Raman anti-Stokes and Stokes signals. The profile shape of the function is mainly determined by the bandwidth performance of the modulation and detection modules in the system.

**Figure 2:**
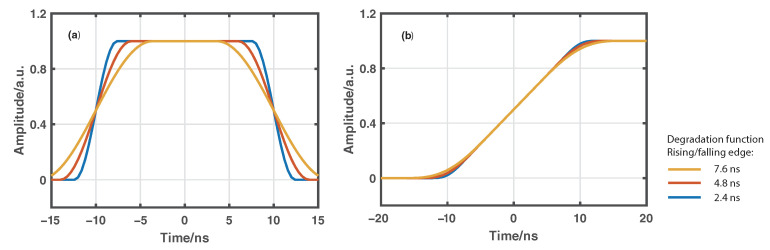
(**a**) Numerical generated degradation functions with the same FWHM of 20 ns but different rising/falling edges of 7.6, 4.8, and 2.4 ns, (**b**) Rising edges of the corresponding simulated Raman signals.

Simulations are conducted to illustrate the relationship between the degradation function profile and the resulting spatial resolution. Figure 2a gives the profiles of three numerically generated degradation functions with the same full-width at half-maximum (FWHM) of 20 ns but three different rising/falling edges controlled by a roll-off factor. According to Equation (3), the Raman signal at the detection end can be simulated through convolutions between the degradation function and a Heaviside function representing the ideal Raman response signal. The rising edge of each convolution result after normalization as shown in Figure 2b is employed to characterize the resulting spatial resolution, which is barely affected by the roll-off factor of the degradation function. The results indicate that the spatial resolution of conventional ROTDR sensor systems is mainly determined by the FWHM of the degradation function dominated by the pump pulse width.

Deconvolution based on Wiener filtering is one of the most classic deconvolution methods to estimate the desired process from the recorded degraded processes with noise. By applying Wiener filtering to the recorded Raman anti-Stokes and Stokes signals as expressed as Equation (3), we can obtain the estimated original Raman signals ${\hat{g}}_{\mathrm{AS}}\left(t\right)$ and ${\hat{g}}_{S}\left(t\right)$ expressed as [30]

(4) $${\hat{g}}_{†}\left(t\right)=F^{-1}\left[\frac{\Psi_{†}^{*}\left(f\right)}{{\left|\Psi_{†}\left(f\right)\right|}^{2}+K}{\tilde{I}}_{†}\left(f\right)\right],$$

where $F^{-1}$ represents the inverse Fourier transform, $\Psi_{†}\left(f\right)$ is the Fourier transform of $\psi_{†}\left(t\right)$, ${\tilde{I}}_{†}\left(f\right)$ is the Fourier transform of recorded Raman signal, and the superscript * donates complex conjugation. *K* is a filtering parameter employed to adjust the filtering bandwidth to achieve a specific spatial resolution. Furthermore, the ratio between the estimated anti-Stokes and Stokes Raman signal given as $A\left(z\right)={\hat{g}}_{\mathrm{AS}}\left(2z/v_{g}\right)/{\hat{g}}_{S}\left(2z/v_{g}\right)$ can be employed to extract the spatial resolution-enhanced temperature distribution via Equation (2).

By substituting the Fourier transform of Equation (3) into Equation (4) and neglecting the noise term, we obtain

(5) $${\hat{g}}_{†}\left(t\right)=F^{-1}\left[\frac{{\left|\Psi_{†}\left(f\right)\right|}^{2}}{{\left|\Psi_{†}\left(f\right)\right|}^{2}+K}\right]*g_{†}\left(t\right),$$

where the equivalent degradation function is defined as

(6) $${\hat{\psi }}_{†}\left(t\right)=F^{-1}\left[\frac{{\left|\Psi_{†}\left(f\right)\right|}^{2}}{{\left|\Psi_{†}\left(f\right)\right|}^{2}+K}\right].$$

This means that the resulting signal after Wiener deconvolution is a convolution of the original ideal Raman response and the equivalent degradation function, which is exactly the Wiener deconvolution result of the original degradation function. According to Equation (6), the profile of the equivalent degradation function and therefore the resulting spatial resolution are determined by the original degradation function and the selection of parameter *K*.

**Figure 3:**
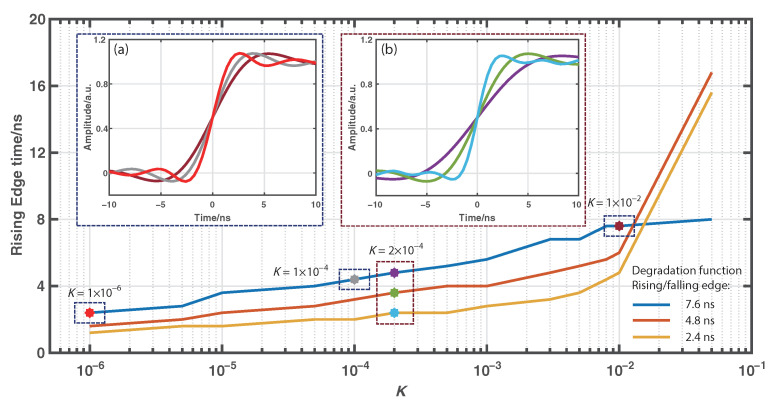
Evolution of the 10/90% rising edge time of simulated deconvoluted Raman signals over parameter *K* for $\psi_{†}\left(t\right)$ with FWHM of 20 ns and rising/falling edges of 7.6, 4.8, and 2.4 ns. Inset: (**a**) Rising edges of the simulated Raman signals for different *K* values and $\psi_{†}\left(t\right)$ with FWHM of 20 ns and rising/falling edge of 7.6 ns. (**b**) Rising edges of the simulated Raman signals for the same hl$K=2\times 10^{-4}$ and $\psi_{†}\left(t\right)$ with FWHM of 20 ns and rising/falling edges of 7.6, 4.8, and 2.4 ns.

Here, simulations have been conducted to investigate the impact of the degradation function profile shape and the Wiener filtering parameter value *K* on the achieved spatial resolution. The three numerically generated degradation functions with different rising/falling edges as shown in Figure 2a are respectively deconvoluted by the Wiener filtering with a selected parameter *K* to obtain their corresponding equivalent degradation functions. The corresponding deconvoluted Raman signal can be simulated through the convolution between the obtained equivalent degradation function and the ideal Raman response signal. The resulting rising edge time is then calculated to examine the achieved spatial resolution. By tuning the value of parameter *K*, we can obtain the evolution of the 10/90% rising edge time of the simulated deconvoluted Raman signals over parameter *K* for $\psi_{†}\left(t\right)$ FWHM of 20 ns and different rising/falling edges as shown in Figure 3. The inset figure (a) of Figure 3 also gives the rising edges of the simulated Raman signals for different *K* values and $\psi_{†}\left(t\right)$ with the FWHM of 20 ns and the rising/falling edge of 7.6 ns. With the decreasing values of *K*, it can be seen that the 10/90% rising edge time decreases to several nanoseconds, indicating the achievement of submetric spatial resolution. In addition, the inset figure (b) of Figure 3 shows the rising edges of the simulated Raman signals for $K=2\times 10^{-4}$ and $\psi_{†}\left(t\right)$ with the FWHM of 20 ns but different rising/falling edges. Under the same value of parameter *K*, a shorter rising/falling edge of the original degradation function leads to a shorter rising edge for the deconvoluted Raman signal and a better spatial resolution for the temperature sensing. It indicates that the bandwidth performance of the modulation and detection modules of the system make great difference on the spatial resolution enhancement of the Wiener deconvolution algorithm.

## 3. Experimental Results

**Figure 4:**
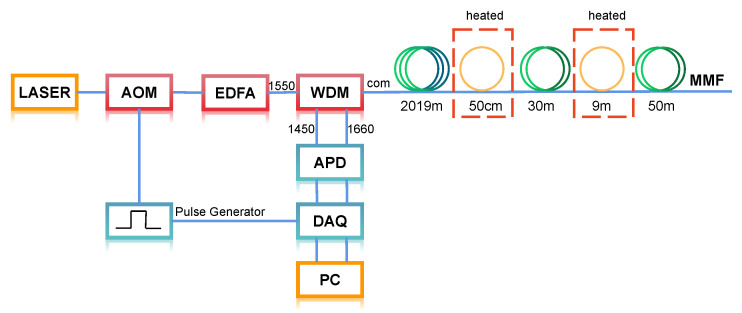
Experimental setup of ROTDR system. AOM, acoustic-optic modulator; EDFA, erbium-doped fiber amplifier; WDM, wavelength division multiplexer; MMF, multimode optical fiber; APD, avalanche photodiode; DAQ, data acquisition card; PC, personal computer.

To validate the enhancement performance of the Wiener deconvolution, experimental demonstrations have been conducted with the experiment setup, as shown in Figure 4. The output of the laser source (1550.12 nm, 2 kHz linewidth) is modulated by an acoustic–optic modulator (AOM, 80 MHz bandwidth) to transform the electrical pulse signal with pulse width of 20 ns to the pulsed pump light. Then, the peak power of the pulses is boosted by an erbium-doped fiber amplifier (EDFA) to about 4 W, which avoids exciting stimulated Raman scattering after guiding it into a multimode fiber (MMF) by a wavelength division multiplexer (WDM). The WDM also acts to guide the spontaneous Stokes and anti-Stokes components backscattered from the COM port to 1660 nm and 1450 nm ports, respectively. The isolation between the ports is over 60 dB, enough to filter out Rayleigh scattering light. The two Raman components are simultaneously detected by a dual-port avalanche photodetector (APD, 500 MHz bandwidth). The output signals are acquired by the two channels via a data acquisition card (DAQ) at a sample rate of 2.5 GSa/s. Finally, the Raman signal is processed with a personal computer (PC). We adopted an averaging of 300,000 for the detected Raman signal traces. The total measurement time is around 36 s, including data collection time around 33 s and signal processing time about 3 s. At around 2 km distance of a 2.1-km MMF under test, two fiber coils of length 50 cm and 9 m spaced by 30 m fiber are heated to 60 °C by a water-bath tank, and the rest of the fiber is stored at 40 °C in a thermostat.

**Figure 5:**
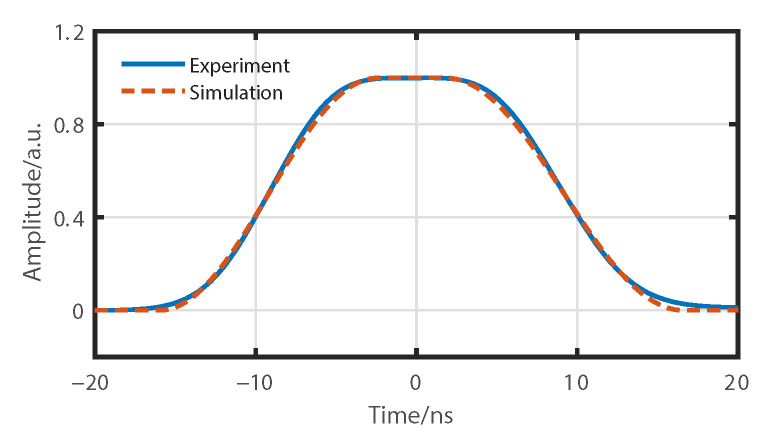
The measured degradation function of a 20-ns pulse for our experimental ROTDR setup (blue curve) and the numerically generated degradation function of 18 ns for simulations (red curve).

According to the analysis in the previous section, the detected pulse profile after normalization (blue curve) as shown in Figure 5 can be employed as the original degradation function of 20 ns for our ROTDR system. We can see the degradation function is not an ideal rectangular profile but a profile with noticeable rising and falling edges, where the FWHM changes to about 18 ns rather than 20 ns. These are mainly due to the limited modulation bandwidth of the AOM in our system.

**Figure 6:**
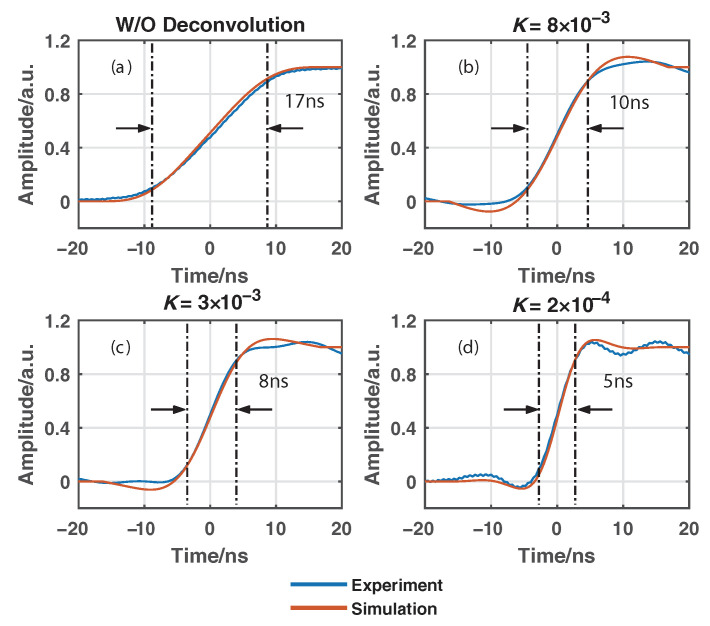
Rising edge of (**a**) the original Stokes Raman signal without using the Wiener deconvolution and the estimated Stokes Raman signals using the Wiener deconvolution with (**b**) $K=8\times 10^{-3}$; (**c**) $K=3\times 10^{-3}$; (**d**) $K=2\times 10^{-4}$. The blue curves are results based on experimental data, and the red curves are simulated results.

**Figure 7:**
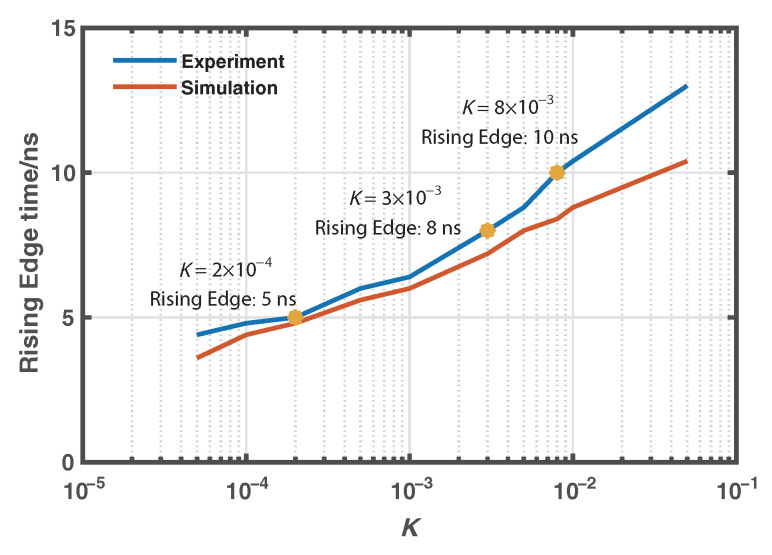
Evolution of the rising edges of the estimated Raman signals based on both experimental and simulated data over parameter *K*.

Algorithms without using and using the proposed Wiener deconvolution are applied to postprocess the received anti-Stokes and Stokes Raman signals, where the convolutions with different *K* values can be efficiently conducted in the frequency domain via fast Fourier transform (FFT). Figure 6 illustrates the rising edges of the measured Stokes signal and the estimated Stokes signals by using the Wiener deconvolution with *K* as $8\times 10^{-3}$, $3\times 10^{-3}$, and $2\times 10^{-4}$, respectively. The 10/90% rising edges of the curves are about 17, 10, 8, and 5 ns, respectively, which means the corresponding spatial resolution is 1.7, 1.0, 0.8, and 0.5 m. In each figure, the rising edge of the corresponding simulated Raman signal based on the numerically generated degradation function given in Figure 5 is also provided in red curve. Figure 7 also gives the evolution of the rising edges of the estimated Raman signals based on both experimental and simulated data over parameter *K*. The results above are normalized for better comparison. We can see that the results based on the experimentally measured data agree well with the simulated ones.

**Figure 8:**
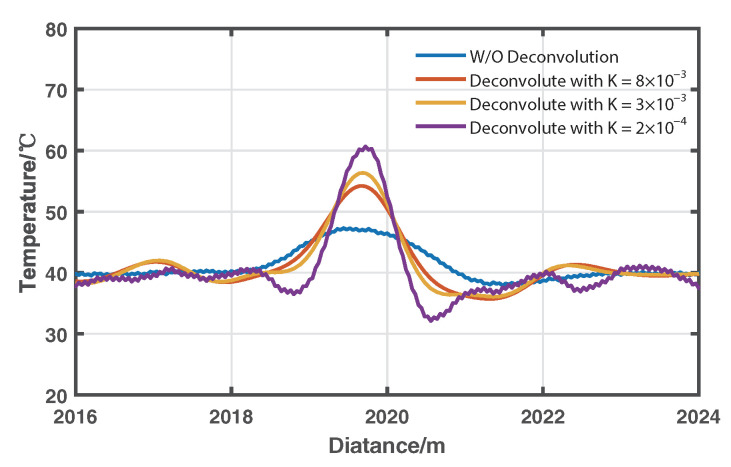
The resolved temperature distributions around the heated 0.5 m fiber coil under different values of parameter *K*.

**Figure 9:**
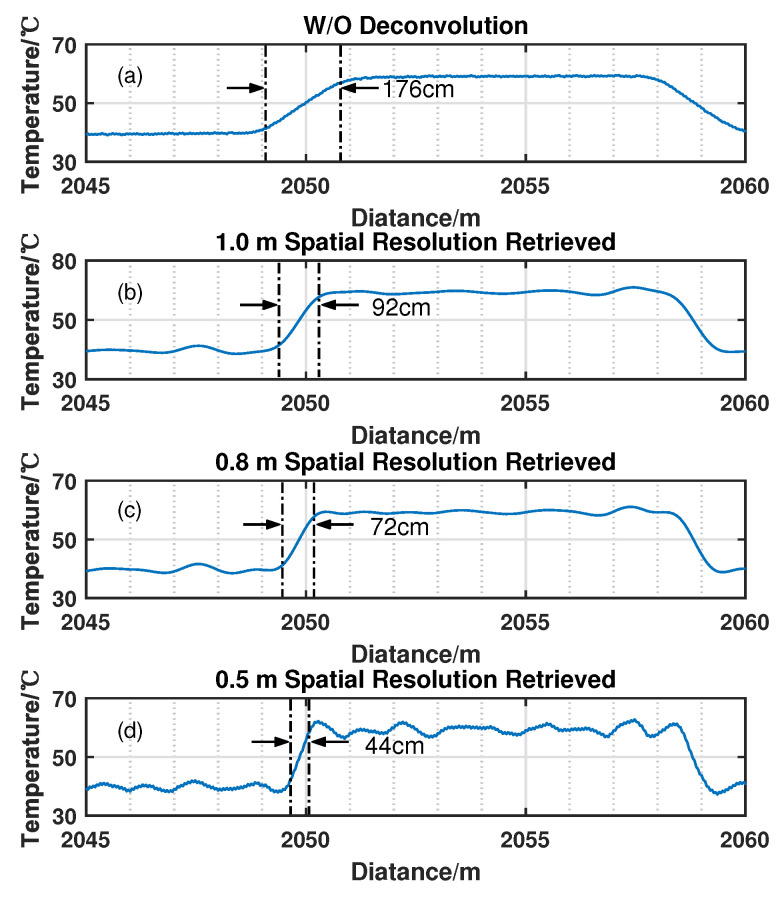
The resolved temperature distributions around the heated 9-m fiber coil (**a**) without deconvolution, (**b**) deconvoluted with parameter $K=8\times 10^{-3}$, (**c**) deconvoluted with parameter $K=3\times 10^{-3}$, (**d**) deconvoluted with parameter $K=2\times 10^{-4}$.

In the demonstration of distributed temperature sensing, it is acceptable to approximate $A_{0}\left(z\right)$ as a constant by calculating the average value of *A* for the last 50-m fiber under the reference temperature $T_{0}$ = 40 °C. The temperature distribution can be calculated based on Equation (2). The spatial resolution enhancement can be verified by the resolved temperature of the heated 0.5-m fiber coil. As we can see from Figure 8, the temperature of the heated 0.5-m coil is correctly resolved as 60.67 °C until *K* equals $2\times 10^{-4}$. Figure 9 gives the resolved temperature distributions of the fiber from 2045 m to 2060 m without using deconvolution and those using Wiener deconvolution with *K* as $8\times 10^{-3}$, $3\times 10^{-3}$, and $2\times 10^{-4}$. The lengths of the temperature transition area are respectively marked as 1.76, 0.92, 0.72, and 0.44 m, which agrees well with the theoretical spatial resolution, as shown in Figure 6. This also approves the spatial-resolution enhancement capability of the algorithm.

## 4. Discussion

**Figure 10:**
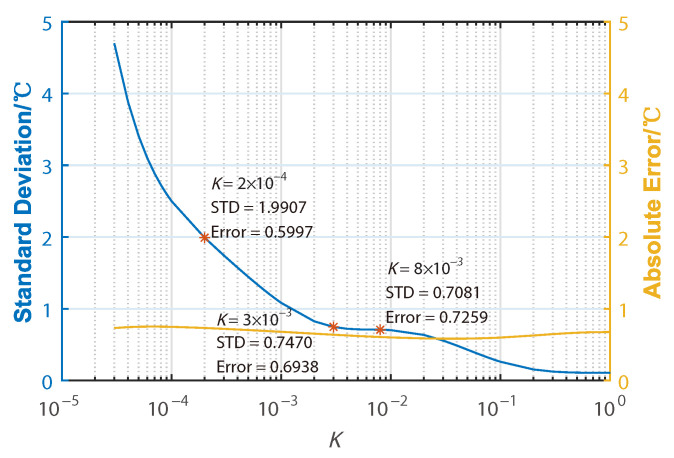
Evolution of the standard deviation and absolute error of the resolved temperature distribution around the heated 9-m fiber coil over parameter *K*.

We have noticed that the introduction of Wiener deconvolution unfortunately also brings in one major impairment for the sensing performance. The noise level of the deconvoluted Raman signal is increasing as parameter *K* decreases, which leads to the sensing accuracy decreasing. However, the absolute error of the detected temperature of the 9-m heated fiber coil is given in Figure 10, which keeps it lower than 1 °C with the decreasing of parameter K. This means that the application of Wiener deconvolution will not affect the temperature sensitivity in ROTDR. As shown in Figure 9a–d and Figure 10, the standard deviations of the resolved temperature of the 9-m heated fiber coil are calculated as 0.20, 0.71, 0.75, and 1.99 °C, respectively, indicating the distortion in restored temperature is also increasing. In practice, to maintain the measurement accuracy, the highest spatial resolution assisted by Wiener convolution is therefore also limited by the original SNR of the acquired signals. For further discussion, we revisit the expression for estimated original Raman signals ${\hat{g}}_{AS}\left(t\right)$ and ${\hat{g}}_{S}\left(t\right)$:

(7) $$\begin{array}{cc} {\hat{g}}_{†}\left(t\right)=F^{-1}\left[\frac{\Psi_{†}^{*}\left(f\right)}{{\left|\Psi_{†}\left(f\right)\right|}^{2}+K}{\tilde{I}}_{†}\left(f\right)\right] & =F^{-1}\left\{\frac{\Psi_{†}^{*}\left(f\right)}{{\left|\Psi_{†}\left(f\right)\right|}^{2}+K}\left[\psi_{†}\left(f\right)g_{†}\left(f\right)+N_{†}\left(f\right)\right]\right\}\\ & ={\hat{\psi }}_{†}\left(t\right)*g_{†}\left(t\right)+F^{-1}\left[\frac{\Psi_{†}^{*}\left(f\right)}{{\left|\Psi_{†}\left(f\right)\right|}^{2}+K}N_{†}\left(f\right)\right] \end{array}$$

where the second term in the equation above contains the noise term $N_{†}\left(f\right)$. This means the noise in the detected Raman signal is also deconvoluted and added in the estimated original Raman signals, leading to the distortion of restored temperature. Therefore, if the SNR is improved with cyclic simplex coding technique [31] or detection components with lower noise level, the temperature distortion will be suppressed. Further analysis of the best spatial resolution assisted by the Wiener deconvolution algorithm will be conducted in our future research work.

Compared to the total variation deconvolution technique for ROTDR sensors as proposed in 2016 [27], the Wiener deconvolution technique has its own advantages. The total variation deconvolution based on the interactive weighted least squares approach was used, where one single iteration requires $9N^{3}+N^{2}$ times of real number multiplication. Although the computational complexity of matrix multiplication and division can be optimized, with the increase in the number of iterations, the higher computational complexity is difficult to meet the timeliness requirements of ROTDR. By comparison, the Wiener deconvolution only requires $4N+4Nlog_{2}N$ times of real number multiplication, which offers obvious advantages. In addition, the temperature calibration process of our scheme is less complex. For our configuration and for conventional ROTDR configuration, temperature demodulation only requires one pair of Raman anti-Stokes/Stokes ratio and temperature at one certain location. By comparison, the total variation deconvolution scheme calculates the impulse response based on the detected temperature profile and the real temperature profile along the entire fiber, and the initial solution needs to be determined to reduce iterations, which makes it less suitable for applications.

## 5. Conclusions

The proposed Wiener deconvolution technique can enhance the spatial resolution of the ROTDR distributed temperature sensor system via a post-processing algorithm. The proposed technique can be applied in existing ROTDR systems without modifying any hardware structure, making it superior in cost and complexity. We have successfully applied the Wiener deconvolution technique in a typical ROTDR system and enhanced the spatial resolution from 2 m to as high as 0.5 m, with a temperature accuracy of 1.99 °C over a 2.1-km MMF. The spatial resolution of the ROTDR system can be further improved with our technique together with other hardware modification or optimization approaches, which makes it promising for precise temperature detection over long distances in the future.

## Data Availability

Not applicable.
